# Slc26a7 Chloride Channel Activity and Localization in Mouse Reissner’s Membrane Epithelium

**DOI:** 10.1371/journal.pone.0097191

**Published:** 2014-05-08

**Authors:** Kyunghee X. Kim, Joel D. Sanneman, Hyoung-Mi Kim, Donald G. Harbidge, Jie Xu, Manoocher Soleimani, Philine Wangemann, Daniel C. Marcus

**Affiliations:** 1 Anatomy & Physiology Department, Cellular Biophysics Laboratory, Kansas State University, Manhattan, Kansas, United States of America; 2 Anatomy & Physiology Department, Cell Physiology Laboratory, Kansas State University, Manhattan, Kansas, United States of America; 3 Department of Medicine and Center on Genetics of Transport, University of Cincinnati, Cincinnati, Ohio, United States of America; Albany Medical College, United States of America

## Abstract

Several members of the SLC26 gene family have highly-restricted expression patterns in the auditory and vestibular periphery and mutations in mice of at least two of these (SLC26A4 and SLC26A5) lead to deficits in hearing and/or balance. A previous report pointed to SLC26A7 as a candidate gene important for cochlear function. In the present study, inner ears were assayed by immunostaining for *Slc26a7* in neonatal and adult mice. *Slc26a7* was detected in the basolateral membrane of Reissner’s membrane epithelial cells but not neighboring cells, with an onset of expression at P5; gene knockout resulted in the absence of protein expression in Reissner’s membrane. Whole-cell patch clamp recordings revealed anion currents and conductances that were elevated for NO_3_^−^ over Cl^−^ and inhibited by I^−^ and NPPB. Elevated NO_3_^−^ currents were absent in *Slc26a7* knockout mice. There were, however, no major changes to hearing (auditory brainstem response) of knockout mice during early adult life under constitutive and noise exposure conditions. The lack of *Slc26a7* protein expression found in the wild-type vestibular labyrinth was consistent with the observation of normal balance. We conclude that SLC26A7 participates in Cl^−^ transport in Reissner’s membrane epithelial cells, but that either other anion pathways, such as ClC-2, possibly substitute satisfactorily under the conditions tested or that Cl^−^ conductance in these cells is not critical to cochlear function. The involvement of SLC26A7 in cellular pH regulation in other epithelial cells leaves open the possibility that SLC26A7 is needed in Reissner’s membrane cells during local perturbations of pH.

## Introduction

The extra-sensory epithelium of the inner ear consists of many cell types that contribute to the maintenance of the unusual composition of the cochlear and vestibular lumen. The luminal fluid, endolymph, is very high in K^+^ (∼150 mM) and low in Na^+^ (1 mM cochlea, 10 mM vestibule) and Ca^2+^ (25 µM cochlea, 250 µM vestibule), a composition that is essential to support transduction of sound and acceleration via the sensory hair cells [1]. It is known that Reissner’s membrane in the cochlea contributes to homeostasis of endolymph by glucocorticoid-regulated absorption of Na^+^ [2], [3], but other potential transport pathways in this epithelium have not yet been determined.

Slc26a7 is a member of the Slc26 transporter family, two others of which have limited expression in the inner ear and which support critical physiological functions. Slc26a4/pendrin mediates HCO_3_^−^ secretion and Slc26a5/prestin is the motor protein that supports outer hair cell electromotility, which is a key element in selectivity and sensitivity of hearing [4]. Knockout of either of these genes in mice leads to deafness [5], [6].

Analysis of gene array data [2] shows a 25-fold higher expression of *Slc26a7* in Reissner’s membrane compared to the neighboring tissue, stria vascularis (Table S1 and Figure S1 in reference [7]) [8], or to kidney [9]. Expression in Reissner’s membrane is also greater than expression in thyroid, which is only 5-fold higher than in stria vascularis or kidney [10].

It has been reported that Slc26a7 functions as a Cl^−^/HCO_3_^−^ exchanger and/or as a Cl^−^ channel [11], [12]. We recently reported that Cl^−^ currents in Reissner’s membrane epithelial cells had composite characteristics consistent with the functional expression of both ClC-2 and Slc26a7 [7]. The highly-limited expression pattern of Slc26a7 and of two other Slc26 transporters in the ear led to the hypothesis that Slc26a7 may be a gene essential for hearing.

The present study sought to test that hypothesis by measuring inner ear expression patterns of Slc26a7, hearing, balance, Cl^−^ conductance properties of Reissner’s membrane epithelial cells and sensitivity to noise exposure in Slc26a7 control and knockout mice. Expression was limited to the basolateral membrane of Reissner’s membrane epithelial cells, and the whole-cell anion conductance exhibited signature properties of Slc26a7 in control epithelia that were absent in cells from knockout mice. No other deficiencies were observed in knockout animals, suggesting that either a compensatory pathway may be expressed in knockout animals or that this conductance is not essential for unstressed inner ear function.

## Results and Discussion

The goal of this study was to determine whether *Slc26a7* is functionally expressed in Reissner’s membrane and whether *Slc26a7* is essential for hearing. We used immunohistochemistry, mouse gene engineering, electrophysiology, as well as hearing and balance tests in this investigation.

### Immunolocalization of Slc26a7

**Figure 1:**
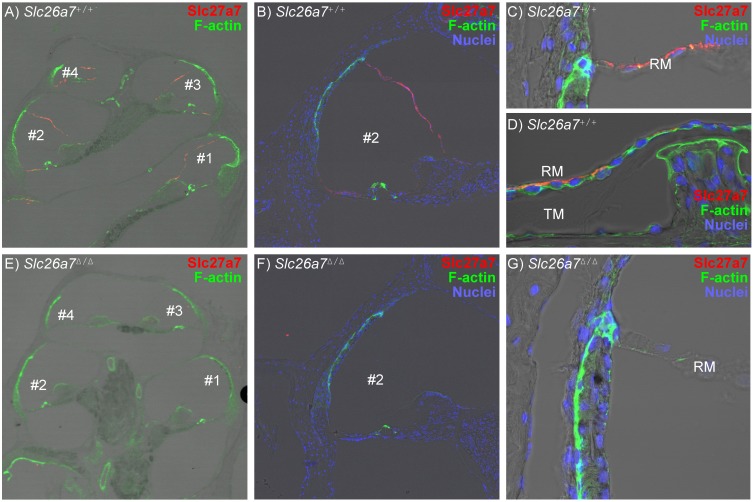
Immunostaining of Slc26a7 in the cochlea. *Slc26a7* ^+/+^ in panels A–D: A) *Slc26a7* was immunolocalized to Reissner’s membrane epithelial cells in a mid-modiolar section of the cochlea of a representative adult *Slc26a7^+/+^* mouse. The four cross-sections of the cochlear duct are labeled in scala media from base to apex. B) Magnified image of cross-section #2. C) *Slc26a7* was observed in Reissner’s membrane (RM) adjacent to the stria vascularis, but expression did not extend into any cells of the lateral wall. The antibody was detected only on the basolateral membrane of the epithelial cells and was not observed in the mesothelial cells. D) *Slc26a7* was observed in Reissner’s membrane near the insertion at the spiral limbus. The antibody was detected only on the basolateral membrane of the epithelial cells and was not observed in the mesothelial cells. E), F), G) No staining was observed in the cochlea of *Slc26a7^Δ/Δ^* mice. Slc26a7, red; F-actin (phalloidin), green; Nuclei (DAPI), dark blue.

**Figure 2:**
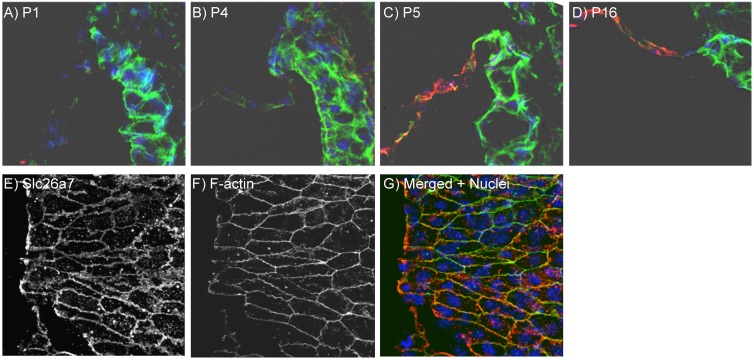
Onset of expression of Slc26a7. A–D) immunolocalization of *Slc26a7* (red) in sections of Reissner’s membrane at the ages P1, P4, P5 and P16. The first expression was detected at P5. E–G) surface preparation of Reissner’s membrane of a P8 mouse stained for Slc26a7 and F-actin. The actin ring is known to associate with the lateral junctions.

Slc26a7 protein expression was immunolocalized to Reissner’s membrane in the cochlea (Figure 1A, B), consistent with the earlier observations of transcript expression via gene array [2] and RT-PCR [7]. No other expression was seen in the cochlea (Figure 1A) and none in the vestibular labyrinth (not shown). Expression in Reissner’s membrane was restricted to the basolateral membrane of the epithelial cells (Figure 1C, D), but staining was absent in *Slc26a7^Δ/Δ^* mice (Figures 1E, F, G). The onset of expression occurred at post-natal day 5 (P5) (Figure 2), which also corresponds to the onset of functional Na^+^ absorption in Reissner’s membrane [13]. Slc26a7 was most strongly observed near the tight junctions when viewed *en face* (Figure 2E–G), likely due to more lateral membrane in the plane of focus compared to the basal membrane.

### Histology

**Figure 3:**
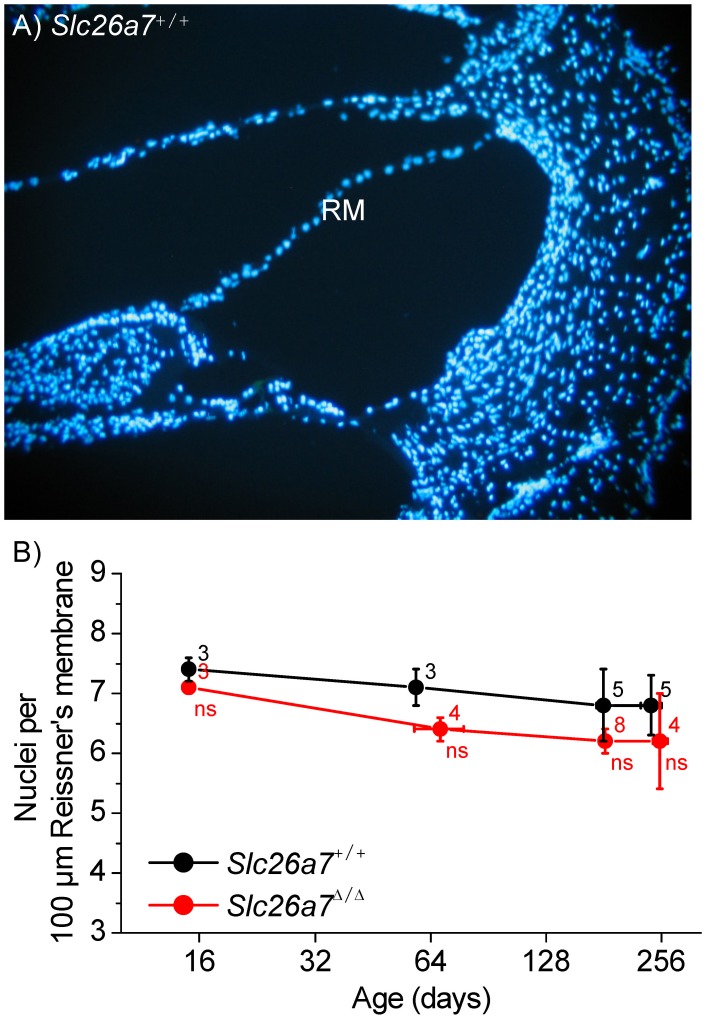
Density of Reissner’s membrane epithelial cells. A) Representative image of mid-modiolar cochlear section of *Slc26a7* ^+/+^ mouse stained with the nuclear dye, DAPI. RM, Reissner’s membrane. B) Summary of counts of nuclei per 100 µm length of Reissner’s membrane. No significant difference (ns) was found in the number of cells per 100 µm between *Slc26a7^+/+^* and *Slc26a7^Δ/Δ^* mice at P15 through 8 months old.

**Figure 4:**
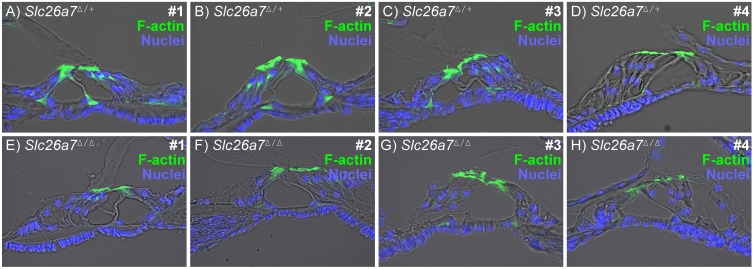
Organ of Corti in *Slc26a7* *^Δ^* ^***/****Δ*^ **and** ***Slc26a7*** *^Δ^* ^***/****Δ*^ Mice. The morphology of mid-modiolar sections of the organ of Corti was compared between heterozygous (*Slc26a7^Δ/+^ top panel*; A, B, C, D) and knockout mice (*Slc26a7^Δ/Δ^, bottom panel*; E, F, G, H) of P15 littermates. No differences were observed between corresponding cochlear half-turns (labeled I, II, III, IV from the base to the apex). An open tunnel of Corti in each turn is consistent with no delay in development at this age.

It was of interest whether deletion of Slc26a7 led to histological changes in the cochlea. Cell density was determined as nuclei per 100 µm length of Reissner’s membrane measured along a mid-modiolar section. There was no loss (Figure 3) of cells in Reissner’s membrane of *Slc26a7^Δ/Δ^* mice at P15. The organ of Corti was normal, including opening of the tunnel of Corti in all four mid-modiolar cochlear half-turns of *Slc26a7^Δ/+^* and *Slc26a7^Δ/Δ^* mice at P15 (Figure 4). Frequency ranges of the four sections were I: 38–46 kHz; II: 19–24 kHz; III: 9–10 kHz; IV: 5–7 kHz, as estimated from [14].

### Transport Gene Expression

**Figure 5:**
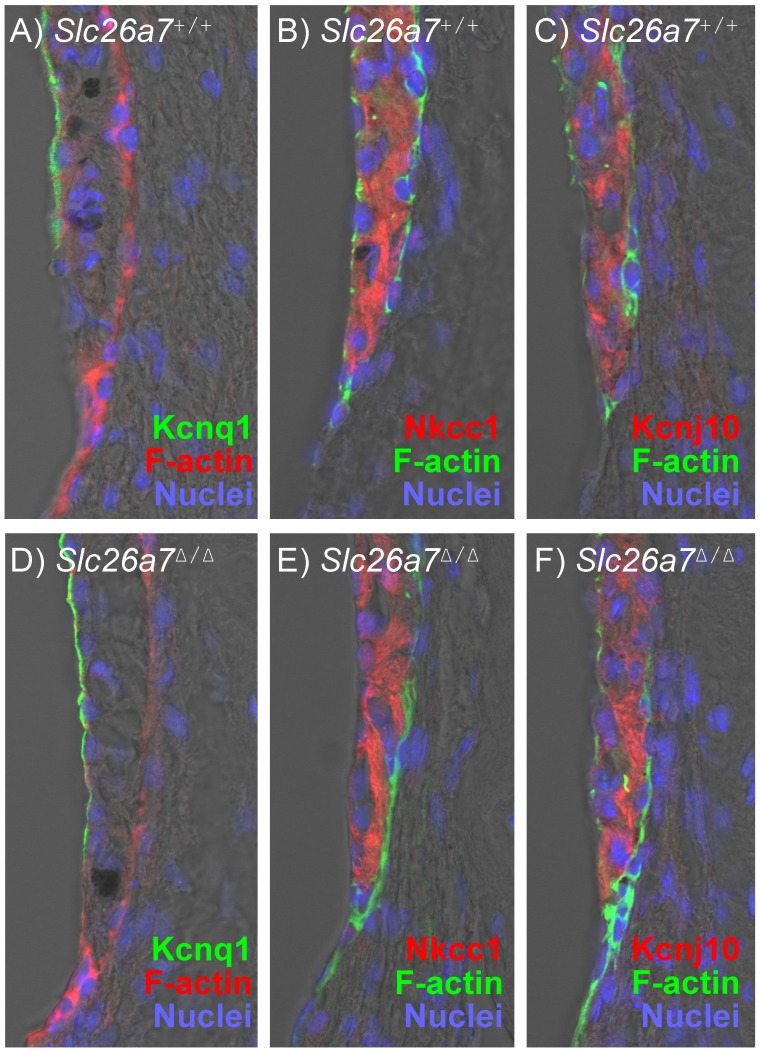
Stria vascularis in *Slc26a7^+/+^* and *Slc26a7^Δ/Δ^* Mice. Expression of key functional transport proteins that are known to be in the stria vascularis and that mediate generation of the endocochlear potential and the high potassium concentration of the luminal fluid, endolymph. There were no differences in the expression between *Slc26a7^+/+^* (*top panel; A, B, C*) and *Slc26a7^Δ/Δ^* (*lower panel; D, E, F*) mice for the potassium channels *Kcnq1* and *Kcnj10* and for the Nkcc1 transporter *Slc12a2*.

Knockout of cochlear transport genes such as pendrin (*Slc26a4*) have been observed to dramatically affect the function and histology of stria vascularis, even though *Slc26a4* is not expressed in the affected cells [15]. By contrast, essential genes for transport of K^+^ and for generation of the transepithelial endocochlear potential (Kcnq1, Kcnj10 and Slc12a2) [16] were observed by immunohistochemistry to be fully expressed in the stria vascularis of both *Slc26a7^+/+^* and *Slc26a7^Δ/Δ^* mice (Figure 5).

### Anion Currents and Conductances

Composite Cl^−^ currents previously observed in Reissner’s membrane epithelial cells had characteristics consistent with mediation by Slc26a7 and ClC-2 anion channels [7]. We sought additional evidence here for functional Slc26a7 channels in Reissner’s membrane epithelial cells. Na^+^ and K^+^ fluxes were excluded from whole-cell patch clamp currents by use of the large cation NMDG^+^ as the sole monovalent cation in the bath and pipette. Whole-cell current and conductance at +80 and +70 mV respectively represent predominantly those of the bath anion inward flux, while those at −80 and −70 mV represent predominantly outward Cl^−^ fluxes supplied by the pipette.

**Figure 6:**
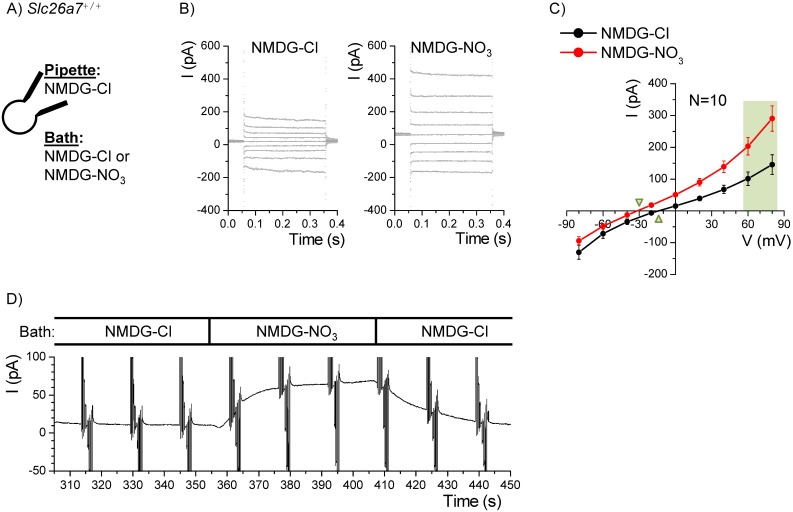
Nitrate currents in Reissner’s membrane epithelial cells from wild-type mice. A) Whole-cell patch clamp currents from Reissner’s membrane epithelial cells from *Slc26a7^+/+^* mice were recorded with Cl^–^-rich pipette solution and either Cl^–^-rich or NO_3_^–^-rich bath solution. B) The command voltage was held at 0 mV and stepped for 300 ms to +80 mV through −80 mV in 20 mV increments. Representative recordings of step responses before *(left)* and at the end *(right)* of the NO_3_^−^ bath perfusion are shown. C) Summary of the mean currents at the end of each step are plotted with SEM error bars. Light green panel shows values used for statistical tests of differences in currents and conductances. Green triangles point to the reversal voltages. D) A representative continuous recording during perfusion of Cl^–^-rich and NO_3_^–^-rich bath solutions at the times marked.

The inward NO_3_^−^ flux in *Slc26a7^+/+^* mice (290.2±40.0 pA) was significantly greater than the inward Cl^−^ flux (145.6±31.3 pA; n = 10) (Figure 6) and similar results were observed in heterozygous *Slc26a7^Δ/+^* mice (n = 2; not shown). In addition, the NO_3_^−^ conductance at +70 mV was significantly greater (4.3±0.6 vs 2.2±0.5 nS) and the reversal voltage (V_r_) was 15 mV more negative than for Cl^−^ (−33.1±2.6 vs −18.0±3.0 mV), consistent with a greater permeability to NO_3_^−^ than to Cl^−^.

**Figure 7:**
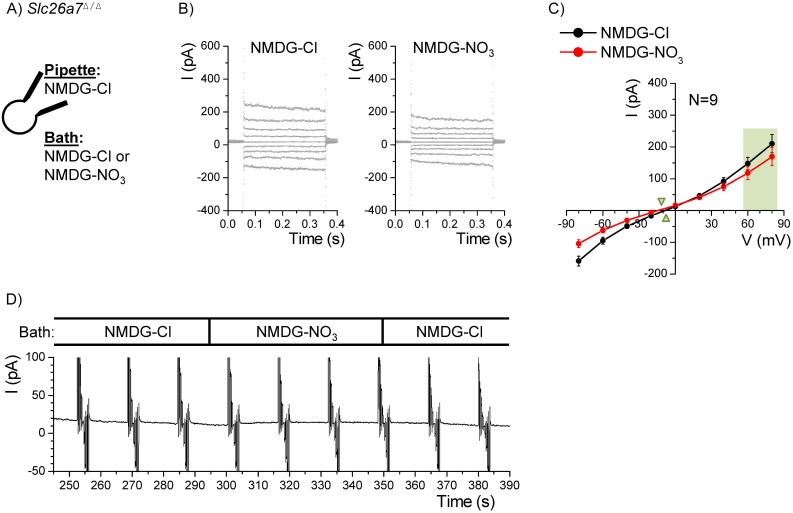
Nitrate currents in Reissner’s membrane epithelial cells from knockout mice. A) Whole-cell patch clamp currents from Reissner’s membrane epithelial cells from *Slc26a7^Δ/Δ^* mice were recorded with Cl^–^-rich pipette solution and either Cl^–^-rich or NO_3_^–^-rich bath solution. B) The command voltage was held at 0 mV and stepped for 300 ms to +80 mV through −80 mV in 20 mV increments. Representative recordings of step responses before *(left)* and at the end *(right)* of the NO_3_^−^ bath perfusion are shown. C) Summary of the mean currents at the end of each step is plotted with SEM error bars. Light green panel shows values used for statistical tests of differences in currents and conductances. Green triangles point to the reversal voltages. D) A representative continuous recording during perfusion of Cl^–^-rich and NO_3_^–^-rich bath solutions at the times marked.

This elevated anion selectivity to NO_3_^−^ was essentially absent in *Slc26a7^Δ/Δ^* mice (Figure 7). The NO_3_^−^ current at +80 mV was actually slightly less than for Cl^−^ (169.5±28.5 vs 210.2±28.8 pA; n = 9). In addition, there was no significant difference in conductance of the bath anions (2.6±0.4 vs 3.1±0.5 nS) although V_r_ was slightly more negative in NO_3_^−^ (−15.4±2.1 vs −10.0±1.8 mV). The origin of this small shift in V_r_ is not clear but is only a third that observed in *Slc26a7^+/+^* mice.

**Figure 8:**
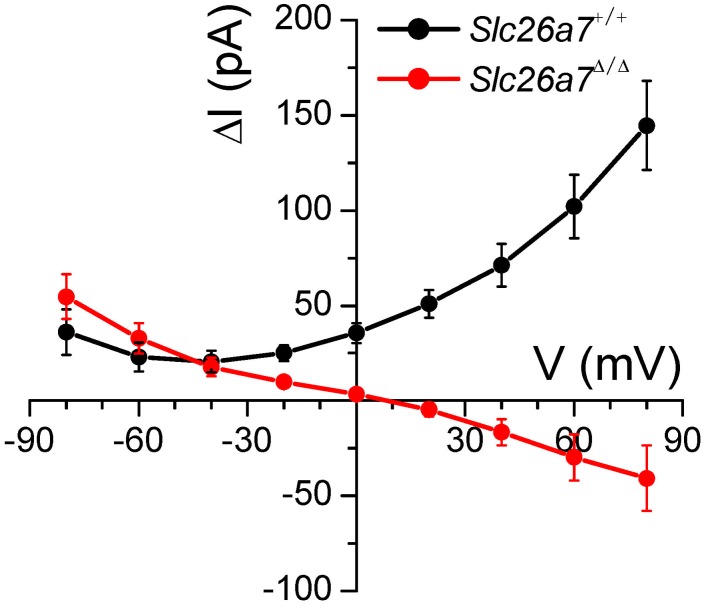
Nitrate currents exceed chloride currents in wild-type but not knockout mice. Currents in NO_3_^–^-rich bath minus currents in Cl^–^-rich bath are plotted for wild-type and for knockout mice. Excess NO_3_^−^ currents (at +80 mV) are significantly greater in wild-type animals, consistent with the contribution of anion currents mediated by *Slc26a7* channels.

The currents summarized in Figures 6C and 7C were further analyzed in Figure 8. The difference between currents measured in NO_3_^−^ bath and Cl^−^ bath are plotted for both *Slc26a7^+/+^* and *Slc26a7^Δ/Δ^* mice. The “excess NO_3_^−^ current” at +80 mV is far higher in cells from *Slc26a7^+/+^* mice than in cells from *Slc26a7^Δ/Δ^* mice. This observation is consistent with the elevated NO_3_^−^ current in wild-type cells being mediated by the *Slc26a7* Cl^−^ channel as reported for heterologously expressed channels [17]. The observation that the excess NO_3_^−^ current becomes negative in the knockout mice suggests that the remaining conductive anion pathways are more permeable to Cl^−^ than to NO_3_^−^. The difference current at −80 mV is about the same in both *Slc26a7^+/+^* and *Slc26a7^Δ/Δ^* mice, as expected since the current was carried predominantly by Cl^−^ in the pipette in both cases.

**Figure 9:**
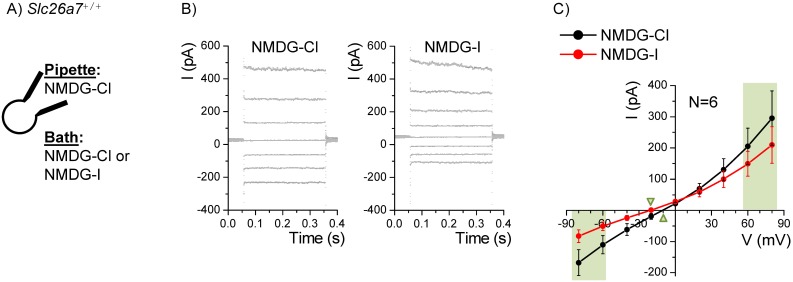
Iodide currents in Reissner’s membrane epithelial cells from wild-type mice. A) Whole-cell patch clamp currents from Reissner’s membrane epithelial cells from *Slc26a7^+/+^* mice were recorded with Cl^–^-rich pipette solution and either Cl^–^-rich or I^–^-rich bath solution. B) The command voltage was held at 0 mV and stepped for 300 ms to +80 mV through −80 mV in 20 mV increments. C) Summary of the mean currents at the end of each step are plotted with SEM error bars. Light green panels show values used for statistical tests of differences in currents and conductances. Green triangles point to the reversal voltages. I^−^ in the bath reduced Cl^−^ efflux current and conductance (at −80 mV) and the I^−^ slope conductance (at +80 mV) was also reduced compared to the Cl^−^ bath, consistent with reduced permeability to I^−^ and partial inhibition of the *Slc26a7* channel.

The effects of bath I^−^ on conductance and current were measured as part of the fingerprint for *Slc26a7* (Figure 9). Both the Cl^−^ conductance (at −70 mV; 1.6±0.3 vs 2.9±0.6 nS; n = 6) and the I^−^ conductance (at +70 mV; 3.0±0.9 vs 4.5±1.5 nS) were significantly reduced in bath I^−^. Further, the Cl^−^ current at −80 mV was significantly reduced by bath I^−^ (−82.6±20.4 vs −167.6±41.4 pA), although the mean I^−^ current at +80 mV did not reach statistical significance compared to the bath Cl^−^ current (209.6±58.6 vs 295.5±87.2 pA).

These changes in conductance by bath I^−^ are consistent with a lower permeability of the *Slc26a7* anion channel to I^−^ than to Cl^−^ and with a partial inhibitory action of I^−^ on anion currents through *Slc26a7*. Both characteristics have been observed in heterologously expressed *Slc26a7* anion channels [17]. The meaning of the lack of significance of the change in current at +80 mV is not clear, but appears to be related to a greater variability in this sample at the positive voltages.

**Figure 10:**
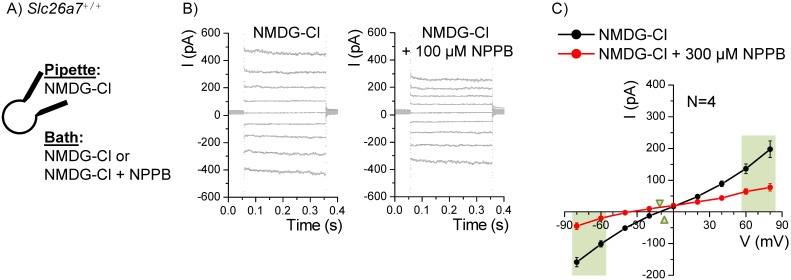
Inhibition of chloride currents by NPPB. A) Whole-cell patch clamp currents from Reissner’s membrane epithelial cells from *Slc26a7^+/+^* mice were recorded with Cl^–^-rich pipette and bath solutions in the absence or presence of NPPB. B) The command voltage was held at 0 mV and stepped for 300 ms to +120 mV through −140 mV in 20 mV increments. Representative recordings of step responses before and at the end of the 100 µM NPPB bath perfusion are shown. C) Currents at the end of each step between +80 and −80 mV are summarized. The currents were inhibited throughout the voltage range and conductances at the light green panels were also reduced.

NPPB (5-Nitro-2-(3-phenylpropyl-amino)benzoic acid) is known to inhibit several anion channels, with a greater potency on Slc26a7 [18] than on ClC-2 [19]. We observed on Reissner’s membrane epithelial cells (Figure 10) that 300 µM NPPB significantly-reduced Cl^−^ currents at all voltages other than zero and that the Cl^−^ conductance at both +70 mV (3.1±0.6 vs 0.7±0.2 nS; n = 4) and at −70 mV (1.9±0.2 vs 1.2±0.2 nS) were significantly decreased. NPPB inhibited Cl^−^ currents at +80 mV with an IC_50_ of 145±43 µM and a Hill Coefficient of 0.5±0.1, fixing maximum inhibition at 100% (Origin software, OriginLab, Northampton, MA, version 7.0). Mouse Slc26a7 expressed in Xenopus oocytes was also sensitive to NPPB [18]. We analyzed their data and obtained a best fit to the Hill equation with an IC_50_ of 38±7 µM and a Hill Coefficient of 1.2±0.2, and an NPPB-insensitive current of 17.6%. By contrast, rabbit ClC-2 is inhibited by NPPB with an IC_50_ of 980±30 µM and a Hill Coefficient of 0.98±0.03 [19]. Our result is consistent with a mixed response to NPPB acting on both Slc26a7 and ClC-2 anion channels, which would be expected to put the IC_50_ between those for the two channels and stretch out the shape of the curve, resulting in an apparent Hill Coefficient less than 1.

Taken together, these observations support the notion that Slc26a7 contributes significantly to the anion conductance of Reissner’s membrane epithelial cells in mice. It is interesting to consider if the contribution of this channel is important under *in vivo* conditions, in view of the strong inward-rectification of the ClC-2-like conductance and the unusual luminal ion composition. The basolateral membrane voltage *in vivo* has not been reported, but one can surmise that it is not highly polarized. Whole-cell patch currents are largest for Na^+^ somewhat smaller for Cl^−^ and comparatively very small for K^+^ [7], [20]. The contribution of Na^+^ conductance to the membrane voltage is likely very little under normal *in vivo* conditions since it is located on the apical membrane where it is in contact on both sides of the membrane with very low [Na^+^] (1 mM in the lumen and presumably about 10 mM in the cytosol). The contribution of K^+^ conductance to the membrane voltage is likely also very little under normal *in vivo* conditions since whole-cell K^+^ currents were found to be very small compared to Na^+^ currents [20]. Although cell membrane voltage was historically thought to be dominated by K^+^ conductance, a substantial number of cells have been found to be dominated by Cl^−^ conductance, including stria vascularis marginal cells in the cochlea and vestibular dark cells [21]–[24]. Since epithelial cells usually have only a moderately lower transmembrane [Cl^−^] difference compared to the [K^+^] difference, the Reissner’s membrane epithelial cells are expected to have a rather moderately-negative voltage.

A hypopolarized negative membrane voltage would not be expected to support large ClC-2 currents. Nevertheless, our results with 300 µM NPPB (Hill coefficient of 0.5), at a voltage (+80 mV) that does not favor ClC-2, suggest substantial action of NPPB on two populations of channel. This is consistent with a significant contribution of ClC-2 to the membrane Cl^−^ conductance under presumed *in vivo* conditions.

Pannexin1 is also an outwardly-rectifying anion channel [25], [26] that is expressed in Reissner’s membrane [2] but not the neighboring stria vascularis [8]. Although the characteristics observed here of outward rectification, greater permeability to NO_3_^−^ than to Cl^−^ [25], and the sensitivity to NPPB [26] are all consistent with the functional presence of pannexin1 [25], [26], the absence of this elevated NO_3_^−^ current in *Slc26a7^Δ/Δ^* mice and the anion permeability sequence of I^−^>Cl^−^ for pannexin1 [25] argues against the observed currents being carried by pannexin1. Taken together, these observations are consistent with anion currents carried specifically by SLC26A7 in Reissner’s membrane epithelial cells.

### Hearing

**Figure 11:**
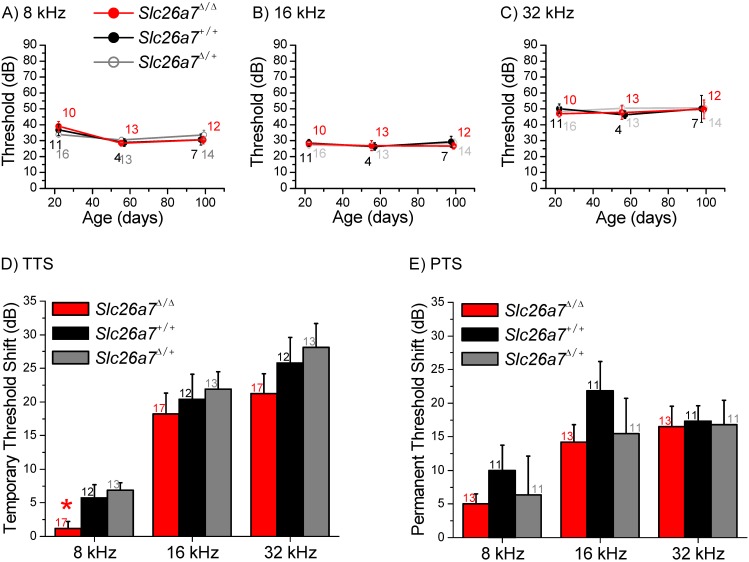
Auditory brainstem recordings. A–C) Auditory brainstem responses (ABR) at 8 kHz, 16 kHz and 32 kHz were made on *Slc26a7^+/+^, Slc26a7^Δ/+^* and *Slc26a7^Δ/Δ^* mice at ages P22 through P100. There were no differences in auditory threshold between either *Slc26a7^Δ/Δ^* or *Slc26a7^Δ/+^* and *Slc26a7^+/+^* mice during their early adult life. The auditory thresholds were unchanged among the genotypes. The numbers of animals for each mean threshold at P22, P56 and P100 are +/+: 11, 4, 7; *Δ/+*: 16, 13, 14; *Δ/Δ*: 10, 13, 12. D) The ABR of *Slc26a7^+/+^, Slc26a7^Δ/+^* and *Slc26a7^Δ/Δ^* mice was measured before and after exposure to noise that produced a temporary auditory threshold shift. E) The ABR of *Slc26a7^+/+^, Slc26a7^Δ/+^* and *Slc26a7^Δ/Δ^* mice was measured before and after exposure to noise that produced a permanent auditory threshold shift. The magnitude of the threshold shifts was not affected by the level of expression of *Slc26a7*. Numbers of animals are given by each bar.

We sought to determine whether SLC26A7 was essential for hearing. Auditory brainstem recordings were made on *Slc26a7^+/+^*, *Slc26a7^Δ/+^* and *Slc26a7^Δ/Δ^* mice during development between P22 and P98 using tone pips at 8, 16 and 32 kHz (Figure 11A, B, C). *Slc26a7^Δ/Δ^* mice were not significantly different from *Slc26a7^+/+^* and *Slc26a7^Δ/+^* mice across these ages. Since the *Slc26a7^Δ/Δ^* mice were not deaf, we hypothesized that an absence of SLC26A7 might potentiate a sensitivity to noise insult. A short, moderate noise exposure caused temporary threshold shifts (TTS) at 3 test frequencies that did not significantly differ among the genotypes in mice between P22 and P98 (Figure 11D), although there was a small, physiologically unimportant protective effect at 8 kHz (Figure 11D). A subsequent more-extreme exposure to noise caused permanent threshold shifts (PTS) that did not significantly differ among the genotypes (Figure 11E). These results suggest that *Slc26a7* is not critical for robustness of hearing.

### Balance

**Figure 12:**
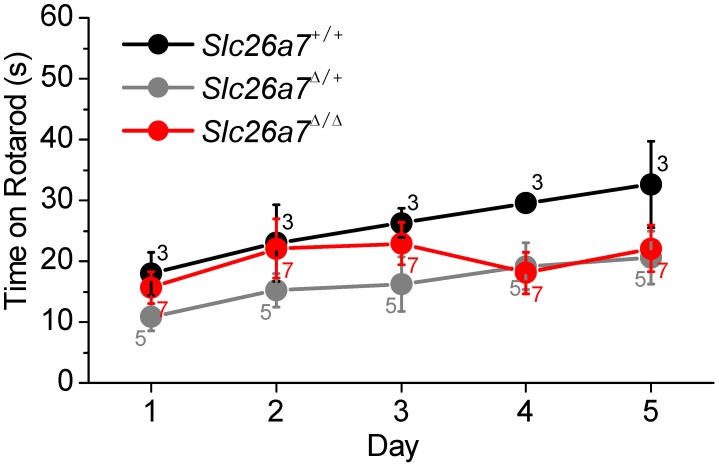
Assessment of balance. *Slc26a7^+/+^*, *Slc26a7^Δ/Δ^* mice and *Slc26a7^Δ/Δ^* mice were tested for balance on a Rotarod apparatus. There were no significant differences in vestibular function among any of the genotypes on any test day (ANOVA).; +/+: n = 3, 2 male, 1 female, 32 g, 10 mo; *Δ/+*: n = 5, 1 male, 4 female, 28 g, 10 mo; *Δ/Δ*: n = 7, 4 male, 3 female, 29 g, 11 mo.

We sought to determine whether SLC26A7 was essential for vestibular function. Vestibular function was tested in knockout *Slc26a7^Δ/Δ^* mice (n = 7) and compared to groups of *Slc26a7^Δ/+^* (n = 5) and *Slc26a7^+/+^* mice (n = 3) using a programmed rotating rod (Figure 12). There were no significant differences in vestibular function among any of the genotypes on any test day., consistent with the absence of immunostaining of SLC26A7 in the wild-type vestibular labyrinth reported above. The mean time for all mice: 20±2 s (n = 15) was comparable to that found in a previous study of Slc26a4 mutant mice with normal vestibular function [27].

## Conclusion

In this study we sought to determine the localization and function of SLC26A7 in the mouse inner ear. The SLC26A7 Cl^−^ channel was localized solely to the basolateral membrane of Reissner’s membrane epithelial cells and shown to be functional in the plasma membrane of those cells. Deletion of that gene from the mouse resulted in a loss of protein expression in Reissner’s membrane and a loss of anion currents carried by that channel. However, there were no major changes to hearing during early adult life under constitutive and noise exposure conditions. The lack of expression found in the wild-type vestibular labyrinth was consistent with the observation of normal balance. We conclude that SLC26A7 participates in Cl^−^ transport in these cells, but other anion pathways such as ClC-2 channels or other channels possibly upregulated in the mutant mice appear to substitute satisfactorily under the conditions tested. The involvement of SLC26A7 in cellular pH regulation in other epithelial cells leaves open the possibility that SLC26A7 is needed in Reissner’s membrane cells during perturbations of pH in either intracellular or extracellular pH.

## Methods

### Ethics Statement

All experiments were conducted on adult mice under a protocol approved by the Institutional Animal Care and Use Committee of Kansas State University (IACUC#2925).

### Mice

*Slc26a7^Δ/Δ^* mice were previously generated and described [28]. Adult mutant mice in a C57BL/6 background (3 wild-type and 3 knockout) were obtained from the University of Cincinnati and were bred at Kansas State University.

### Immunohistochemistry and Confocal Microscopy

Mice were anesthetized with 4% tribromoethanol (0.014 ml/g body wt i.p.) and transcardially perfused with phosphate-buffered saline (PBS) solution (4 ml, 1 min) followed by 4% paraformaldehyde in PBS (12 ml, 3 min). Temporal bones were removed and locally perfused with 4% paraformaldehyde in PBS from the round and oval windows to the apex, 5 min at 11 µl/min each and then decalcified in 10% EDTA in PBS 24 hrs at 4°C with rocking. Decalcified cochleae were cryoprotected by sequential exposure to increasing sucrose concentrations in PBS (10% for 30 min, 20% for 30 min and 30% overnight) and infiltrated with a 2∶1 mixture of Tissue Freezing Medium (Electron Microscopy Sciences, Hatfield, PA) with 30% sucrose in PBS. Cryosections (10 µm, CM3050S, Leica, Nussloch, Germany) were blocked with 10% bovine serum albumen in PBS with 0.15% Triton-X (PBS-TX).

Slides were treated with primary antibody for Slc26a7 (1∶30) [29] in PBS-TX with 1% BSA and were incubated overnight at 4°C. The Alexa Fluor 594 goat secondary anti-rabbit antibody (1∶200, Molecular Probes, Eugene, OR) was incubated for 1 h at 25°C. The other primary antibodies used were goat anti-Kcnq1 1∶200 (C20, Santa Cruz Biotechnology, Santa Cruz, CA), rabbit anti-Kcnj10 1∶300 (Alomone, Jerusalem, Israel) and rabbit anti-Slc12a2 1∶100 (Chemicon, Temecula, CA) and incubated with appropriate secondary antibodies, as before [30].

Alexa Fluor 488 phalloidin (1∶40, Molecular Probes Inc.) and DAPI (1∶300, Molecular Probes Inc.) were co-stained with the antibodies. Images of slides mounted with FluorSave (Calbiochem, La Jolla, CA) were obtained by confocal microscopy (LSM 510 Meta, Carl Zeiss, Göttingen, Germany). Laser-scanning brightfield images were obtained for visualizing structure and a Zeiss Axiocam (model MRm; to increase depth of field) with mercury light source was used to capture images of DAPI staining of nuclei for counting cell density in Reissner’s membrane (Figure 3). Statistical significance was evaluated using unpaired t-tests and a significance criterion of P<0.05.

### Auditory Brain Stem Recordings and Noise Exposure

Mice were anesthetized with 4% tribromoethanol (0.014 ml/g body wt i.p.) following a protocol approved by the Institutional Animal Care and Use Committee of Kansas State University and auditory brainstem recordings (ABR) were obtained as previously described [27]. Calibrated tone-burst stimuli (21/s) were delivered with a free-field electrostatic speaker and stimuli were 2 ms duration, 0.5 ms gate time at 8, 16 and 32 kHz at alternating phase between 90 and 10 db SPL with 10 db intervals. Responses were recorded over 10 ms and filtered below 300 and above 3000 Hz plus a 60 Hz notch filter, and 1000 sweeps were averaged. Auditory thresholds were determined by visual comparison of the averaged waveforms.

Sensitivity of hearing to noise exposure was tested by obtaining an initial ABR as above, followed by exposure to Gaussian noise (14 to 18 kHz) at a level of 109 dB for either 10 s (to produce temporary threshold shift) or 2 h (to produce permanent threshold shift). A post-noise ABR was obtained beginning about 2 min after the 10 s noise exposure and 2 weeks after the 2 h noise exposure. The noise signal was generated with Tucker Davis Technologies RPvdsEx software. Statistical significance between knockout and control mice was evaluated using unpaired t-tests and a significance criterion of P<0.05.

### Tissue Preparation and Electrophysiology

Mice were anesthetized with 4% tribromoethanol (0.014 ml/g body wt i.p.). During deep anesthesia, mice were decapitated following a protocol approved by the Institutional Animal Care and Use Committee of Kansas State University. Both temporal bones were collected from each mouse and Reissner’s membrane was isolated and mounted in a perfusion chamber as previously described [2], [3], [7]. Whole-cell patch clamp methods were also as previously described [7]. The whole-cell patch clamp configuration was chosen since the Slc26a7 channels are located on the inaccessible basolateral membrane (*vide infra*) and since single-channel recordings have not been reported for this channel.

The intracellular solution consisted of (in mM) 150 N-methyl D-glucamine (NMDG)-Cl, 10 HEPES, 0.273 CaCl_2_, 1 MgCl_2_, 1 EGTA, pH 7.3. The extracellular solution contained (in mM) 150 NMDG-Cl, 10 HEPES, 0.7 CaCl_2_, 1 MgCl_2_, 5 glucose, pH 7.3. All chemicals were purchased from Sigma and all experiments were performed at 37°C. Whole-cell currents were recorded using pCLAMP 9 software (clampex 9, Axon Instruments) with an Axopatch 200A amplifier (Axon Instruments, Foster City, CA), low-pass filtered at 1 kHz and digitized at 5 kHz with a Digidata 1322A (Axon Instruments) data acquisition system. Concurrent continuous traces were recorded using AxoScope software (Axon Instruments) with a MiniDigi 1A data acquisition system (Axon Instruments) and digitized at 1 kHz. Pipette resistances were 4.5–6.3 MΩ (n = 20). Liquid junction potential was nearly zero because of symmetrical NMDG-Cl in intracellular and initial extracellular solutions. Statistical significance was evaluated using the paired t-test to compare the effects of solution changes, the unpaired t-test to compare genotypes and a significance criterion of P<0.05.

Whole-cell recordings were attributed to single epithelial cells within the tissue, as there is no evidence for coupling among the epithelial cells nor between epithelial cells and mesothelial cells. Electron microscopic images show no gap junctions between the epithelial cells and none between epithelial and mesothelial cells, which are separated by a basement membrane [31], [32]. Further, preliminary measurements of whole-cell currents were not substantially changed upon perfusion of either of the gap junction blockers 18β-glycyrrhetinic acid (100 µM) or octanol (2 mM) (KX Kim and DC Marcus, unpublished).

Cell capacitance of Reissner’s membrane epithelial cells was in the range 11.6–25.5 pF for wild-type mice and 14.0–17.0 pF for Slc26a7 knockout mice; there was no significant difference between the two groups (wild-type: 18.4±0.6, n = 26; knockout: 15.4±0.4, n = 9). The seemingly-large range of capacitance values is consistent with the observation of variable sizes of the epithelial cells in the tissue (*vide infra*, Figure 2F) and with the whole-cell capacitance of guinea pig Reissner’s membrane [33].

### Vestibular Function Test

*Slc26a7^+/+^*, *Slc26a7^Δ/+^* mice and *Slc26a7^Δ/Δ^* mice were tested 4 times/day over five days. The RotaRod (Series 8, IITC, Woodland Hills, CA) used a 32 mm diameter rod and was programmed to start at 4 RPM, increasing to 40 RPM over 1 minute. All mice were placed on the rod before rotation was initiated and the times that the mice remained on the rotating rod were recorded. Each mouse was tested four times, three minutes elapsed from start to start of each run and the four observations for each mouse were averaged to obtain the daily mean time. The daily mean time for each mouse was used to calculate the mean and standard error of each genotype on each day. Statistical significance was determined using one-way analysis of variance (ANOVA) on each day.
